# Reproducibility and interobserver agreement of the R.E.N.A.L. nephrometry score: focus on imaging features

**DOI:** 10.1590/0100-3984.2015.0174

**Published:** 2017

**Authors:** Richard Mast Vilaseca, Antonio Carlos Westphalen, Henrique Ferreira Reis, Orlando Salomão Zogbi, Gyl Eanes Silva, Rodolfo Borges dos Reis, Valdair Francisco Muglia

**Affiliations:** 1 MD, Attending Radiologist, Radiology Department - Abdominal Imaging, Hospital Universitari Vall d'Hebron, Barcelona, Spain.; 2 MD, PhD, Associate Professor of Radiology, Radiology and Biomedical Engineering, University of California at San Francisco (UCSF), San Francisco, CA, USA.; 3 MD, Attending Radiologist, Internal Medicine Department - Imaging Division, Hospital das Clínicas da Faculdade de Medicina de Ribeirão Preto da Universidade de São Paulo (FMRP-USP), Ribeirão Preto, SP, Brazil.; 4 MD, PhD, Assistant Professor, Department of Pathology, Faculdade de Medicina de Ribeirão Preto da Universidade de São Paulo (FMRP-USP), Ribeirão Preto, SP, Brazil.; 5 MD, PhD, Assistant Professor, Department of Surgery - Urology Division, Faculdade de Medicina de Ribeirão Preto da Universidade de São Paulo (FMRP-USP), Ribeirão Preto, SP, Brazil.; 6 MD, PhD, Associate Professor, Department of Radiology, Faculdade de Medicina de Ribeirão Preto da Universidade de São Paulo (FMRP-USP), Ribeirão Preto, SP, Brazil.

**Keywords:** Carcinoma, renal cell, Kidney neoplasms, Neoplasm staging, Computed tomography, Magnetic resonance imaging

## Abstract

**Objective:**

To investigate the reproducibility and interobserver agreement for R.E.N.A.L. nephrometry scoring system.

**Materials and Methods:**

Two independent radiologists retrospectively analyzed 46 consecutive patients with renal masses, between 2008 and 2012, using the R.E.N.A.L. nephrometry score (RENAL-NS), which is based on the evaluation of five anatomical features of the tumor, as evaluated with computed tomography or magnetic resonance imaging: *R*adius, *E*xophytic/endophytic properties, *N*earness to the collecting system, *A*nterior or posterior descriptor, and *L*ocation relative to the polar line. Tumor complexity was graded as low, intermediate, or high. The interobserver agreement was calculated for the total score and for the score for each parameter. Surgical excision of the tumors was used as the standard of reference.

**Results:**

The interobserver agreement for each of the RENAL-NS parameters, respectively, a hilar location, and the total score was 98%, 80%, 100%, 89%, 85%, 89%, and 93% of patients, corresponding to kappa values of 0.96, 0.65, 1.00, 0.75, 0.72, 0.78, and 0.88, respectively. The Nearness, Radius, and total score showed the best agreement. For the cases that were discordant in terms of the final score, no major implications in surgical planning were observed.

**Conclusion:**

The RENAL-NS is a structured, useful system to assess the anatomical features of renal tumors. It is easily applicable and reproducible, even for less experienced radiologists.

## Resumo

**Objetivo:**

Investigar a concordância interobservador e a reprodutibilidade do sistema de pontuação nefrométrico R.E.N.A.L. nephrometry score (RENAL-NS) usando imagens de tomografia computadorizada e ressonância magnética.

**Materiais e Métodos:**

Dois observadores independentes analisaram, retrospectivamente, 46 pacientes com massas renais, consecutivos, de 2008 a 2012. Cinco características anatômicas foram avaliadas: raio (R); localização exofítico/endofítico (E); proximidade do tumor ao sistema coletor (N, *nearness*); posição anterior (A) ou posterior e de localização em relação à linha polar (L). Os tumores foram classificados em baixo, intermediário ou alto grau de complexidade. A concordância foi calculada para cada parâmetro do escore e para o valor global. A excisão cirúrgica das lesões foi utilizada como padrão de referência.

**Resultados:**

A concordância para cada um dos parâmetros renais, para a característica de "h", e para a pontuação final foi 98%, 80%, 100%, 89%, 85%, 89% e 93% dos pacientes, o que corresponde a valores de kappa de 0,96, 0,65, 1,00, 0,75, 0,72, 0,78 e 0,88, respectivamente. Os parâmetros "N", "R" e o escore global mostraram as melhores concordâncias. Entre os casos discordantes na pontuação final, não foram observadas grandes mudanças no planejamento cirúrgico.

**Conclusão:**

O RENAL-NS é um sistema estruturado, útil para avaliar características anatômicas do tumor renal. É de fácil aplicação, reprodutível, mesmo entre os radiologistas menos experientes.

## INTRODUCTION

A steady increase in the incidence of kidney cancer has been seen in the last two decades^(1,2)^, and it is now the seventh most commonly diagnosed cancer in Western countries^(3)^. Nevertheless, mortality has remained stable or even declined over the same time frame^(4)^. In the United States, for example, kidney cancer represents the thirteenth leading cause of cancer death and only approximately 25% of patients die from the disease^(3)^. Such paradox is at least in part attributed to the widespread use of imaging techniques for scanning abdomen, which allows for early detection and treatment of cancer^(2,4,5)^.

Imaging plays an important role not only in the diagnosis of these tumors, most of which are renal cell carcinomas, but also in treatment decision-making^(6,7)^. Cross-sectional imaging modalities allow an accurate assessment of the location of the tumor and its relationship with the adjacent structures and uninvolved renal parenchyma, and this information is of utmost importance for planning surgical and ablative therapies.

**Figure 1:**
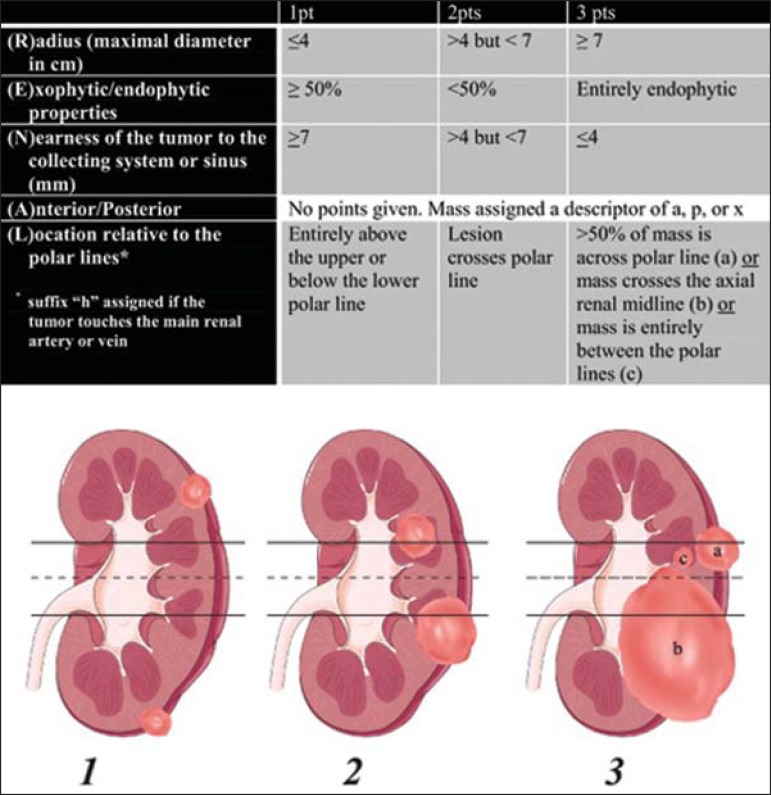
R.E.N.A.L. nephrometry score with scoring of (L)ocation component. Polar lines (solid lines) and axial renal midline (broken line) are depicted on each sagittal view of kidney. Numbers 1 to 3 represent points attributed to each category of tumor. Reproduced of Kutikov and Uzzo^(10)^ (with permission).

Although several different classification models have been proposed to classify renal tumors^(8,9)^, they have achieved limited success in reliably and consistently characterizing tumor anatomy. Recently, however, a new system has been proposed to characterize the anatomical complexity of renal masses and to standardize the description of anatomical features. Kutikov and Uzzo^(10)^ proposed the use of the R.E.N.A.L. nephrometry score (RENAL-NS) system to describe tumors systematically using reproducible and pertinent features: *R*adius (maximal diameter); *E*xophytic/endophytic properties; *N*earness to the collecting system or renal sinus; *A*nterior or posterior descriptor; and *L*ocation relative to the polar lines (Figure 1).

Other authors have evaluated the reproducibility of the RENAL-NS^(11-13)^, its applicability and reproducibility by radiologists. However, there is still a need for external validation and assessment of reproducibility by radiologists with different levels of skill. Accordingly, we conducted this study to assess the reproducibility of and interobserver agreement for the RENAL-NS system in a cohort of patients who underwent surgical treatment.

## MATERIALS AND METHODS

### Patients

This was a retrospective, single-institution study, approved by the Institutional Review Board and Research Ethics Committee of the University of São Paulo at Ribeirão Preto School of Medicine Hospital das Clínicas, in the city of Ribeirão Preto, Brazil. The need for informed consent was waived. From July 2008 to July 2012, 102 consecutive patients with a diagnosis of a renal mass were identified in the kidney mass database of the institution. The inclusion criteria were as follows: having presented with a single renal mass suggestive of renal cell carcinoma; having undergone multidetector computed tomography (MDCT), magnetic resonance imaging (MRI), with images in the axial and coronal planes and volumetric acquisition; and having undergone open or laparoscopic nephrectomy (partial or total). Patients for whom surgical or pathological confirmation was unavailable were excluded (*n* = 20), as were those for whom the images (including high-resolution coronal images) were suboptimal for analysis (*n* = 26), those who presented with multiple lesions (*n* = 6), and those who presented with renal anomalies (malrotation, pelvic kidney, horseshoe kidney, or crossed fused ectopia) or other anatomical variants (*n* = 4). Therefore, the final study sample comprised 46 patients.

### Imaging technique

For the CT examinations, we used a 16 multidetector CT (Brilliance; Philips, Best, the Netherlands) or a 64 MDCT (Somatom Sensation; Siemens, Erlangen, Germany). All studies involved pre-contrast and dynamic post-contrast acquisition (arterial, venous, and equilibrium phases), synchronized using the bolus tracking technique for the arterial phase, then a 30-s delay for the venous phase, and a 90-s delay for the equilibrium phase.

We obtained MRI scans in a 1.5 T, 16-channel scanner (Achieva; Philips, Best, the Netherlands), using a dedicated phased-array body coil in the following sequences: axial and coronal T2-weighted fast spin-echo, with a repetition time/echo time (TR/TE) of 5122/74 ms; axial T1-weighted, inphase, spoiled gradient echo (SGE), with a flip angle of 80° and a TR/TE of 140/4.5 ms; and out-of-phase SGE, with a flip angle of 80° and a TR/TE of 140/2.2 ms. Post-contrast images were acquired with fat suppression and a volumetric gradient-echo breath-hold T1, with the same delays as CT, after intravenous injection of gadopentetate dimeglumine (Magnevist; Belex Laboratories, Wayne, NJ, USA).

### Image evaluation

Two radiologists (HFR and OSZ), one a third-year resident and the other a radiologist with 8 years of experience in abdominal imaging, independently evaluated and scored all lesions. Both radiologists were blinded to patient management and outcomes. Each lesion was scored using the RENAL-NS system^(10)^. A three-point scale was used for each R.E.N.A.L. component except for "A", to which we added the suffix "a" for the anterior location, "p" for posterior location, and "x" when the location was indeterminate. In addition, the suffix "h" was used in order to designate a hilar location if the tumor abutted the main renal artery or vein (Figure 1). After all of the points had been summed, tumors were classified as low-risk (4-6 points), intermediate-risk (7-9 points), or high-risk (10-12 points).

### Surgical treatment

One of the authors (VFM) independently reviewed the kidney cancer database, medical charts, and other records in order to retrieve all of the surgical data.

All procedures were performed by a urologist (RBR) with more than 15 years of experience. Surgeons were free to perform the surgical procedures according to their own expertise and on the basis of any intraoperative findings, assuming the procedures were in accordance with the recommendations set forth in the European Association of Urology guidelines^(14)^. The mean interval between imaging and surgery was 68 days (range, 1-284 days).

### Histological assessment

All surgical specimens were processed at our facility. A standard protocol was followed, and all cases were analyzed by a uropathologist (GES) with 11 years of experience. The same uropathologist retrospectively reviewed all pathology reports and available slides for the purposes of this study. The anatomical and pathological features of all lesions were collected and recorded in a Microsoft Excel spreadsheet (Microsoft Excel for Mac 2011, version 14.3.6; Microsoft, Redmond, WA, USA).

### Statistical analysis

Kappa statistics was used in order to assess interobserver agreement of for the final total RENAL-NS system scores^(15)^. Kendall's coefficient of concordance was used for the ordinal variables. Kappa values can range from 0 to 1, the former indicating a lack of agreement and the latter indicating perfect agreement, respectively. Agreement was considered slight at values = 0.20, fair at values from 0.21 to 0.40 fair, moderate at values from 0.41 to 0.60, substantial at values from 0.61 to 0.80 high, and almost perfect at values ≥ 0.81.

## RESULTS

The demographic and histopathological data are shown in Table 1. The mean patient age was 59.3 ±12.2 years, ranging from 28 to 81 years. Of the 46 patients evaluated, 32 (69.5%) were male.

**Table 1:** Demographic and histopathological data.

Variable	N = 46
Age, in years, mean (range)	59.3 (28–81)
Gender	
Male, *n* (%)	32 (69.5)
Female, *n* (%)	14 (30.5)
Lesion size, in cm, mean (range)	5.4 (1.8–14.2)
RENAL-NS class	
Low complexity, *n* (%)	9 (19.5)
Intermediate complexity, *n* (%)	12 (26.0)
High complexity, *n* (%)	25 (54.5)
Pathological staging of primary malignancies (*n* = 42)	
T1a, *n* (%)	10 (23.8)
T1b, *n* (%)	15 (35.7)
T2, *n* (%)	6 (14.3)
T3, *n* (%)	11 (26.2)
Final diagnosis	
Benign, *n* (%)	3 (6.5)
Metanephric adenoma, *n*	1
Angiomiolipomas, *n*	2
Malignant, *n* (%)	43 (93.5)
Clear-cell RCCs, *n*	29
Papillary RCCs, *n*	7
Chromophobe RCCs, *n*	5
Mucinous tubular and spindle cell RCC, *n*	1
Metastasis from colon cancer, *n*	1

RCC, renal cell carcinoma.

**Figure 2:**
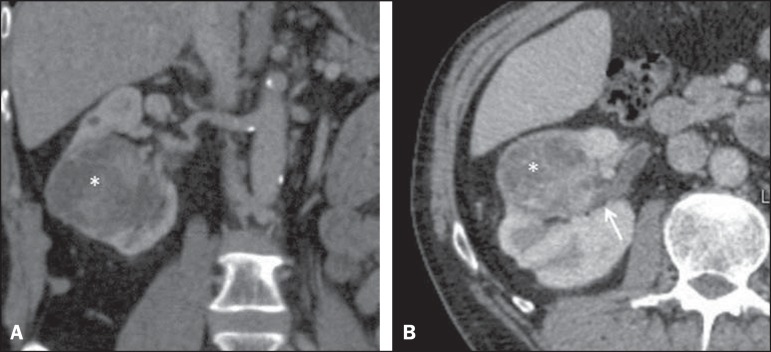
Coronal (**A**) and axial (**B**) CT images showing a complex mass (asterisk in **A** and **B**) in the middle of right kidney, invading renal sinus and extending to renal vein (arrow in B). A 8ah mass of moderate complexity, proved to be a clear cell renal cell carcinoma, pT3bNoMo.

**Figure 3:**
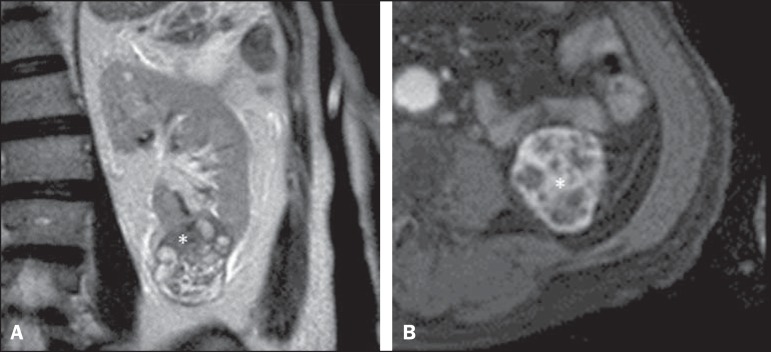
Coronal T2-weighted (**A**) and axial post-contrast T1-weighted (**B**) MR images demonstrate a lower pole, heterogeneous mass in left kidney, predominantly exophytic. After removal, a clear cell renal cell carcinoma was confirmed, pT1aNoMo, classified as low complexity according to RENAL-NS.

Twenty-five patients (54.4%) underwent MRI. Lesions were located in the right kidney in 26 patients (56.5%). The lesions were benign in three patients (6.5%), of whom one had a metanephric adenoma and two had lipid-poor angiomyolipomas (Figure 2). In the remaining 43 subjects (93.5%), the lesions were malignant: one was a metastasis from colorectal cancer, and 42 were renal cell carcinomas (Figure 3). The mean longest axis of the tumors was 5.4 cm, ranging from 1.8 to 14.2 cm.

The interobserver agreement for each R.E.N.A.L. component score was 97.8% (45/46), 80.4% (37/46), 100% (46/46), 89.1% (41/46), and 84.8% (39/46), respectively. Agreement for the "h" parameter was 89.1% (41/46).

The total score indicated that the complexity of the lesions was low in 9 patients (19.6%), intermediate in 12 (26.1%), and high lesions in 25 (54.3%). For the overall classification of lesions, the interobserver agreement was 91.3% (42/46). In two cases, reader 1 classified the lesions as being of moderate complexity (scores of 9 and 7), whereas reader 2 classified the same lesions as being of high and low complexity (scores of 10 and 6), respectively. In two other cases, reader 1 classified the lesions as being of high complexity (a score of 10 for both) and reader 2 classified the lesions as being of moderate complexity (a score of 9 for both). The total score was the same for both observers in 33 patients (71.7%). In 12 cases (26.1%), there was a 1-point difference, and in one case (2.2%), there was a 2-point difference. These results and the corresponding kappa values for each of the R.E.N.A.L. component scores, the "h" parameter, and the total score are summarized in Table 2.

**Table 2:** Frequency of exact agreement between the two reviewers and corresponding kappa values for the scoring components

Statistic	R	E	N	A	L	h	Total
Agreement (%)	98	80	100	89	85	89	93
Kappa value	0.96	0.65	1.00	0.75	0.72	0.78	89
p-value	0.03	0.1	0	0.1	0.09	0.09	0.06
95% CI	(0.9–1.03)	(0.44–0.85)	(1)	(0.55–0.96)	(0.54–0.91)	(0.59–0.96)	(0.77–1.01)

95% CI, 95% confidence interval.

Table 3 summarizes interobserver agreement and reproducibility of the RENAL-NS system from this and previous studies^(11-13)^.

**Table 3:** Comparison of results of published series to assess interobserver agreement and reproducibility of the RENAL-NS system.

		Montag	Kolla	Weight	Present
Variable		et al.^(11)^	et al.^(12)^	et al.^(13)^	study
Patients, *n*		149	51	95	46
Readers, *n*		2	3	6	2
R component	% agreement	96	94	-	98
	Kappa	0.90	0.95	0.87	0.96
					
E component	% agreement	92	94	-	98
	Kappa	0.9	0.95	0.87	0.96
					
N component	% agreement	86	66	-	100
	Kappa	0.86	0.76	0.61	1
					
A component	% agreement	96	80	-	89
	Kappa	0.95	0.84	0.56	0.75
					
L component	% agreement	89	54	-	85
	Kappa	0.85	0.73	0.70	0.72
					
h parameter	% agreement	99	88	-	89
	Kappa	0.94	0.84	0.57	0.78
					
Total score	% agreement	74	82	-	93
	Kappa	0.85	0.80	0.75	0.88

## DISCUSSION

Our findings demonstrate substantial to almost perfect agreement for the individual component and total RENAL-NS system scores between two radiologists. The best results were found for tumor size (*R*adius) and *N*earness to the collecting system. This is important as many consider tumor size a key feature for planning surgical resection of kidney tumors^(16,17)^, and proximity to the collecting system may be a good predictor of complications of nephron-sparing surgery^(18,19)^. In a recent study, the RENAL-NS system was applied to determining outcomes of percutaneous radiofrequency ablation and cryoablation of renal tumors^(20)^. The results of that study showed a good correlation between the total RENAL-NS system score and the treatment results, including the probability of complications. In particular, the data suggest that tumor size and location (anterior or posterior) are important predictors, facilitating the choice between open and laparoscopic procedures.

Our data are in keeping with those of previous studies in terms of the reproducibility of the RENAL-NS system, with only slight variations in the categories^(11-13)^. The frequency of T3 lesions in our sample (25%) was higher than that reported in previous studies—12% in Weight et al.^(13)^, 4% in Kolla et al.^(12)^, and 2.7% in Montag et al.^(11)^—indicating that our patients had lesions that were more complex.

It is of note that we also found almost substantial agreement for parameters prone to subjective interpretation, such as the "E" component, albeit inferior to that observed for the other parameters. In addition our results for the "E" and "L" components are similar to those reported by Montag et al.^(11)^ and Kolla et al.^(12)^.

Although highly complex lesions predominated, being identified in 25 (54.5%) of the 46 lesions evaluated in the present study, lesions of low and moderate complexity were well represented in our sample—in 9 (19.5%) and 12 (26%), respectively—suggesting that the RENAL-NS system shows good reproducibility and agreement, regardless of the complexity of the lesion.

Although many factors probably influence agreement in image interpretation, it is likely that an appropriate scanning technique is one of the most important elements. Scans should be performed in accordance with carefully planned protocols. All of the CT and MRI scans included in our study were enhanced with intravenous contrast media, and enhancement was evaluated in three phases: the corticomedullary phase, to assess arterial anatomy; the nephrographic phase, to define the contours of the neoplasm and its location in the kidney^(21,22)^; and the delayed phase, to assess the "N" component and the "h" parameter. The combination of nephrographic and delayed phase evaluation seems the best approach to evaluate the "N" and "L" components. In addition, volumetric acquisitions were available for all selected patients, and that allowed us to make multiple-plane isotropic reconstructions, which are essential for the correct use of the RENAL-NS system^(23)^. Although not systematically investigated in this research, oblique planes proved extremely valuable for accurately defining the relationship between the tumor and the hilar vessels.

It should be noted that other systems for the anatomical characterization of renal tumors have been proposed^(8,9)^. The preoperative aspects and dimensions used for anatomical (PADUA) classification is based on seven scoring items and focuses on tumor geometry^(8)^. In contrast, the centrality index (C-index) has been described as a method of quantifying the nearness of neoplasms to the renal sinus^(9)^. Both systems have been submitted to internal and external validation^(24,25)^. The RENAL-NS system incorporates features of both of those systems and could therefore be a more robust system^(26,27)^. Albeit a relevant question, we did not evaluate the efficacy of the RENAL-NS system for predicting the type of surgery; nor did we determine whether the surgical planning was carried out according to the RENAL-NS prediction. In a recent study, Okhunov et al.^(28)^ compared the PADUA, C-index, and RENAL-NS systems. The authors found that the level of interobserver agreement was high for all three systems.

Montag et al.^(11)^ proposed a modification to the RENAL-NS system; that is to assign points to the "h" and "A" parameters in order to have a totally numeric classification. Although that is an interesting and perhaps desirable proposal, it will require further examination, not only to determine the reproducibility but also to assess how well the modified system predicts treatment outcomes.

Our study has several limitations. First, due to its retrospective nature, biases may have been introduced. Selection bias is an example, because subjects in our study were required to have undergone surgery. It is possible, therefore, that the mean lesion size in our sample was larger than that in the general population, smaller lesions being more likely to be managed with active surveillance or focal ablative therapy. If that was the case, our results may not be fully generalizable to other populations. Arguably, imaging heterogeneity is also a result of a retrospective design, and we compared data from CT and MRI in the present study. However, the RENAL-NS system assesses only morphological features, and the specific analyses of density and signal intensity are not included in the score system. In addition, our CT and MRI protocols are standardized and were not modified during the study period. Furthermore, we opted to use only two independent reviewers. However, one reader was a junior radiologist (a third-year resident) and our data suggest that the RENAL-NS system is easy assimilated by the novices in the field. Lastly, the number of subjects included in this study is not large and this led to wide 95% confidence intervals around some of our estimates.

In conclusion, the RENAL-NS system is an applicable and reproducible system for evaluating the anatomical characteristics of renal tumors. In our study, the best interobserver agreement was observed for tumor size and nearness to the collecting system.
